# Sexual violence and sub-Saharan migrants in Morocco: a community-based participatory assessment using respondent driven sampling

**DOI:** 10.1186/1744-8603-10-32

**Published:** 2014-05-08

**Authors:** Ines Keygnaert, Abdessamad Dialmy, Altay Manço, Jeroen Keygnaert, Nicole Vettenburg, Kristien Roelens, Marleen Temmerman

**Affiliations:** 1 ICRH- Faculty of Medicine & Health Sciences, Ghent University, De Pintelaan 185 UZP114, Ghent 9000, Belgium; 2 Faculty of Letters and Human Sciences, University Mohammed V, 34 rue Sebou Agdal, Rabat 10090, Morocco; 3 Institute of Research, Training and Action on Migration (IRFAM), 17 Rue Agimont, Liège 4000, Belgium; 4 Department of Social Welfare Studies, Ghent University, H. Dunantlaan 2, Ghent 9000, Belgium

**Keywords:** Sexual violence, Rape, Prevention, Migrants, Morocco, Community Based Participatory Research (CBPR), Respondent Driven Sampling (RDS), European Neighbourhood Policy (ENP)

## Abstract

**Background:**

The European Union contracted Morocco to regulate migration from so-called “transit migrants” from Morocco to Europe via the European Neighbourhood Policy. Yet, international organisations signal that human, asylum and refugee rights are not upheld in Morocco and that many sub-Saharan migrants suffer from ill-health and violence. Hence, our study aimed at 1) investigating the nature of violence that sub-Saharan migrants experience around and in Morocco, 2) assessing which determinants they perceive as decisive and 3) formulating prevention recommendations.

**Methods:**

Applying Community-Based Participatory Research, we trained twelve sub-Saharan migrants as Community Researchers to conduct in-depth interviews with peers, using Respondent Driven Sampling. We used Nvivo 8 to analyse the data. We interpreted results with Community Researchers and the Community Advisory Board and commonly formulated prevention recommendations.

**Results:**

Among the 154 (60 F-94 M) sub-Saharan migrants interviewed, 90% reported cases of multiple victimizations, 45% of which was sexual, predominantly gang rape. Seventy-nine respondents were personally victimized, 41 were forced to witness how relatives or co-migrants were victimized and 18 others knew of peer victimisation. Severe long lasting ill-health consequences were reported while sub-Saharan victims are not granted access to the official health care system. Perpetrators were mostly Moroccan or Algerian officials and sub-Saharan gang leaders who function as unofficial yet rigorous migration professionals at migration ‘hubs’. They seem to proceed in impunity. Respondents link risk factors mainly to their undocumented and unprotected status and suggest that migrant communities set-up awareness raising campaigns on risks while legal and policy changes enforcing human rights, legal protection and human treatment of migrants along with severe punishment of perpetrators are politically lobbied for.

**Conclusion:**

Sub-Saharan migrants are at high risk of sexual victimization and subsequent ill-health in and around Morocco. Comprehensive cross-border and multi-level prevention actions are urgently called for. Given the European Neighbourhood Policy, we deem it paramount that the European Union politically cares for these migrants’ lives and health, takes up its responsibility, drastically changes migration regulation into one that upholds human rights beyond survival and enforces all authorities involved to restore migrants’ lives worthy to be lived again.

## Abstract (fr)

**Résumé:**

**Contexte**

Dans le cadre de sa politique de voisinage, l’Union Européenne tente, en partenariat avec le Maroc, de réguler la migration irrégulière de « transit ». Toutefois, les organisations humanitaires signalent que de nombreux migrants subsahariens souffrent de violence et d’une mauvaise santé. Notre étude vise à 1) étudier la nature des violences que les migrants subsahariens subissent au et dans le voisinage du Maroc, 2) en identifier les déterminants tels que perçus par les migrants, 3) formuler des recommandations de prévention.

**Méthodes**

En appliquant des méthodes de recherche participatives, nous avons formé douze migrants subsahariens en tant que chercheurs communautaires afin de conduire des entretiens approfondis, en utilisant la technique d’échantillonnage dirigé par les répondants. Nous avons analysés les données avec Nvivo8. Les résultats ont été interprétés avec le conseil communautaire du projet. Des recommandations de prévention ont été formulées.

Parmi les 154 (60 F – 94H) migrants subsahariens interrogés, 90% ont signalé des cas de victimisation multiple, la moitié à caractère sexuel, principalement des viols collectifs. Au total, 89 répondants ont personnellement été victimes, 41 ont été forcés d’assister aux violences subies par leurs parents ou compagnons de voyage et 18 ont rapporté des situations vécues par des pairs. Les victimes subissent les séquelles de ces violences sur le long terme, alors qu’elles n’ont pas accès au système de santé marocain. Les auteurs des violences sont essentiellement des fonctionnaires, ou des chefs de gangs qui contrôlent les points de frontière. Ils semblent procéder en toute impunité. Les témoins lient les facteurs de risque notamment à leur situation irrégulière et à leur manque de protection.

Au Maroc, les migrants subsahariens sont à haut risque de victimisation sexuelle. Il est urgent de mettre en œuvre des actions transfrontalières de prévention à divers niveaux. Compte tenu de sa politique de voisinage, nous estimons que l’Union Européenne doit se soucier des menaces encourues par ces migrants. Elle doit prendre ses responsabilités et changer sa politique migratoire afin de respecter elle-même les droits fondamentaux et la dignité de ces groupes, ainsi que d’inciter l’ensemble des autorités concernées à faire de même.

## Background

### Migrants in Morocco

In 2008 as in 2005, Morocco estimated that around 60.000 foreigners were regularly residing on its territory, predominantly from European (47%) and Algerian (19%) origin [1,2]. In addition, official estimates of sub-Saharan irregular migration in Morocco varied between 10.000 in 2005 [1]; 15.000 in 2007 [2]; and 4.500 in 2010 [3] for a total population of nearly 32 million. The sub-Saharan migrants present in Morocco in 2008 originated of about 40 different countries with the most numerous being from Nigerian (15.7%), Malian (13.1%), Senegalese (12.9%) and Congolese (10.4%) origin [4]. The vast majority of them were male (79.7%) and relatively young (95.4% under the age of 36) [4], were employed in their countries of origin (76%) and more than half (56%) had completed secondary or higher education [5]. Estimates of their dispersion in Morocco in 2008, indicated that about 3000 sub-Saharan migrants were living in Rabat, 2000 in Casablanca, 600 in Oujda and Laayoune and 300 in Tangiers [4].

### Migration to Morocco

Sub-Saharan migrants use different lengthy and predominantly over-land routes to get to Morocco [6]. On these routes, a stretched sub-Saharan migration network has arisen and a number of cities, such as Agadez (Niger), Nouadhibou (Mauritania) or Tamanrasset (Algeria), have become ‘migration hubs’ or ‘turntables’ for migrants [4,7], fragmenting their journeys [8]. A large majority eventually passes through Algeria and crosses the cities of Ghardaïa and Maghnia which face Oujda on the Algerian-Moroccan border [4]. Migrants with sufficient financial means pay to be smuggled along the migration networks [7]. The men are frequently asked to pay their fee fully upfront, while women only have to pay a small first instalment or are asked to pay later on [9]. Some, especially Nigerian women, know before departure they will pay through sex work with law enforcement agents or other key people along the route. Others are trapped into fierce sexual exploitation through debt bondage with the young ones, again frequently Nigerian, eventually destined to European sex industry [9-11]. The poorer sub-Saharan migrants who cannot afford a full-package smuggling, try to meander along the routes by the little means they have, fragmenting their journeys at the ‘migration hubs’ at longer interval [7,9]. Both groups make use of the technological developments in the Saharan and Sahel regions of cheap mobile phone and e-mail access, money transfer facilities [12] but also of the ambiguous social and economic relationships that are being formed with those who assist or accompany them during their journey [9]. Both at the ‘hub’ cities as at the Algerian-Moroccan borders there are migrants who organized themselves into ‘migration professionals’ assisting, managing, controlling and/or exploiting the migrants on the way. Especially at the Algerian-Moroccan border, which was closed between 1994 and 2005 following diplomatic tensions [1], these men have a strong reputation of operating in gangs led by chairmen, robbing and attacking the migrants in impunity as official authority is lacking [9,11,12].

### Migration policy

The legal framework around migration in Morocco is recent and is mostly determined by the law 02–03, which was passed in 2003 along with the creation of a dedicated department within the Home Affairs Ministry, and which entered into force in 2010. The provisions of this legal document have been widely debated by NGOs and human rights advocates. They emphasized that the law is poorly known by authorities and that it contains provisions criminalizing migrants. Although it theoretically provides a frame protecting some migrant groups, such as pregnant women or minors, and limiting refoulement to the borders, those provisions do not appear to be thoroughly implemented yet [13,14]. Moreover, the law 02–03 established significant fines for Moroccans helping undocumented migrants and providing them with transportation, a measure that has encouraged a number of transporters to systematically discriminate against Sub-Saharans to avoid being accused of smuggling migrants [13]. Finally, the provisions do not contain clear distinctions between the different migrant groups – refugees, asylum seekers and undocumented – and consequently fuel uncertainties and violations of rights in the field [1]. In addition, although Morocco has signed the Geneva Convention in 1956, UNHCR documents are not yet fully acknowledged by Moroccan security forces [15] and a 2010 assessment of UNHCR action in Morocco emphasized the limited progress the Moroccan state had made to protect refugees and asylum seekers [16].

### The European neighbourhood policy

The relationship between Morocco and the European Union is administered though the European Neighbourhood Policy (ENP), which was launched in 2004 with the ambition of tightening relationships with the neighbouring countries in order to increase prosperity, security and stability [17]. The topic of migration has regularly sparked tensions between the two partners. In 2000, the EU initiated negotiations aiming at reaching an agreement on the readmission by third countries of their own nationals and all other persons having transited through their territories to reach Europe [5]. In 2005, the EU was also encouraging the implementation of ‘transit centres’ in third countries, where asylum candidates could pursue the procedure before entering the European territory, a project that was fiercely condemned by Morocco [5]. The ENP Strategy Paper drafted for the 2007–2013 period emphasized the principle concern of illegal migration for cooperation between Morocco and the EU [18]*.* Between 2007 and 2010, Morocco received a total of €654 million within the ENP framework [17], with at least €130 million in the first two years specifically dedicated to migration [19] through various instruments and notably development aid [13].

### Morocco: a transit country to Europe?

Both EU and Moroccan migration policies are based on the rationale that Sub-Saharan migrants enter and cross Morocco in the hope of reaching Europe, over land to the Spanish enclaves of Ceuta and Melilla in Morocco, or by boat, hidden in vehicles, false visas or other means to the European main land. Consequently, Morocco, along with other countries, was labelled in the mid-1990s as a ‘transit country’ or the ‘migration hub to Europe’ [20]. However, research has shown that not all sub-Saharan migrants in North Africa are on their way to Europe [21]. In contrast, an increasing number of them settle in Morocco whether by primary choice or by default [22] as their journey is rarely planned from one fixed starting to another fixed end point and as failures along their journey might limit future options and drain resources [12,21]. A numerous group among them are thus rather ‘stranded migrants’ than ‘transit migrants’ enhancing their vulnerability and protection needs [12]. The concept of ‘transit migration’ is however used by both the EU, which hence justifies its predominant role in the definition of migration policy in its neighbourhood [23]; and by Morocco, for which it provides a leverage for negotiations with the EU and for reinforcing its military presence in Western Sahara [22]. As a result, border controls have become increasingly militarized [24], making migrants’ living conditions more precarious without succeeding at reducing migration flows [13].

The role of the EU in the development of migration legislation and policy in Morocco is thus to be thoroughly questioned. Co-operation between the EU and third countries in the field of migration has become a major policy focus and “one of the strategic priorities in the external relations of the Union” [25] since the 1999 Tampere Council [23]. Research has consistently addressed this question and many critics have been raised towards this so-called externalization of EU migration policies [24]. The concept of externalization conveys the idea that the EU seeks to delegate its responsibilities in terms of border control to non Member States and uses international cooperation to restrict population movements [13]. Morocco has been widely used in the literature as a meaningful example of border control externalization. The relationship between the two partners has been rather unstable, with tensions in the early 2000s over the negotiation of a readmission agreement [23]. The diversity of agendas within the EU, the attempts of Morocco to divert attention from undocumented Moroccan migrants [24], and a distorted understanding of Sub-Saharan migration patterns are listed among the main explanatory factors. The fact that sub-Saharan migrants travel mostly over land within Africa has largely been attributed to the tightening of European borders and the externalization of migration policies [6,12].

### Sexual violence

Sexual violence refers to “use of physical force to compel a person to engage in a sexual act against his or her will, whether or not the act is completed, an attempted or completed sex act involving a person who is unable to understand the nature or the condition of the act, to decline participation or to communicate unwillingness to engage in the sexual act (e.g. because of illness, disability, or the influence of alcohol or other drugs, or due to intimidation or pressure)” [26]; it is “made by any person regardless of their relationship to the victim, in any setting.” [27]. Based on the level of physical contact we can distinct 4 forms of sexual violence: sexual harassment (no physical contact); sexual abuse (physical contact-no penetration); attempted and completed rape or forced sodomy (physical contact with penetration in body opening) [28]. Within (forced) migration, sexual exploitation, forced prostitution and sexual violence as a weapon of war or torture are considered as extra types of sexual violence” [29]. Refugees, asylum seekers and undocumented migrants are considered to be at high risk of sexual victimization worldwide [30]. Research on sexual violence in these types of migrants in other countries demonstrated the frequent co-occurrence of sexual violence with physical, emotional and socio-economic forms of violence [31,32], which is commonly referred to as gender-based violence in refugee, conflict and humanitarian settings [29,33,34]. Sexual and gender-based violence in migrants can have severe and long-lasting negative repercussions on the victims’ sexual, reproductive, physical and mental health and well-being as well as participation in society [29,31-37].

For Morocco, a national hotline report of 2009 showed that 12, 3% of the reported cases regarded women being raped by non-partners and 1.4% by partners. Sexual harassment accounted for 8.2% and incest for 9,6% of the reported cases to these hotlines [38]. A national report of the same year gave lifetime sexual victimization rates for Moroccan women of 22,6% and an annual rate of 8.7% [39]. This annual rate of 8.9% of violence cases that were reported to authorities in the last 12 months being sexual violence, has been found consistent since 2008 [40]. Migrants are not included in national surveys, yet NGO’s emphasize in their reports that migrant women are sexually exploited and brutally raped both during migration, at the borders as well as in Morocco, which often results in severe health threats [3,4,41]. Furthermore, they registered cases where migrants were denied the lodging of a complaint and/or were brought back to the border [13].

### Research objective

Given their global vulnerability to sexual violence [29-37], the fact that Morocco is contracted by the EU to regulate irregular migration from Morocco to Europe [17,18] and that international organisations signal that asylum and refugee rights are not upheld in Morocco [15], we assume that undocumented migrants might be at risk of serious ill-health and violence in Morocco and at its borders. Hence, our study aimed firstly at getting an idea of the nature and magnitude of violence that sub-Saharan migrants experience in Morocco and on their way to it; secondly, to assess which determinants the migrants perceive as decisive in victimization and prevention; and thirdly, which recommendations can be formulated for comprehensive prevention.

## Methods

### Community based participatory research

Our epistemology stems from an interpretive, feminist, communitarian and dialogical research perspective [42,43]. As this epistemology favours participatory research, we adopted the qualitative and collaborative research approach of Community-Based Participatory Research (CBPR) right from the initial idea to write a project proposal on this topic onwards. CBPR in public health focuses on social, structural, physical and environmental inequalities and aims to improve the health and well-being of community members by a) setting up structures for participation of the target communities, civil society, NGO’s, global institutions and organisations, policy makers and researchers concerned by the research topic, and this in all phases of the project, and by b) integrating knowledge in action, including social and policy change [44,45]. In a first phase in 2008, a participatory partnership was created, comprising of a Scientific Advisory Board (SAB) and a Community Advisory Board (CAB). The purpose of this participatory partnership was to meet at regular interval, to decide mutually on processes and procedures, and to interpret results of steps taken before proceeding to a next phase. The SAB comprised of the Belgian and Moroccan coordinators, Belgian and Moroccan academic experts on migration, violence and sexual health; students mentored by the coordinators and experts on these matters and eventually the community researchers. The CAB consisted of key people in sub-Saharan communities present in Morocco, representatives of migrant associations, civil society, Moroccan and international organisations assisting migrants in Morocco, Moroccan organisations working on human rights, sexual health promotion and violence prevention and policy makers. Along the project, more and more organisations joined the CAB. In order to facilitate knowledge sharing and joined decision-making, several communication strategies were simultaneously adopted. This ranged from regular phone calls, mails, SAB and CAB meetings to detailed newsletters being issued every six months and public participatory seminar at the end of the project in Morocco. After having created this participatory partnership, the conceptual framework and study design was jointly decided upon. The conceptual framework combined the socio-ecological framework [46] on (sexual) health and violence [31], with the concept of Desirable Prevention. Desirable Prevention seeks to improve the health and well-being of all through the concurrent application of five dimensions, being: integrality; participation; inclusiveness; addressing root causes and maximizing agency [47]. The study design intended to integrate the conceptual framework with a thorough application of the CBPR approach resulting in a process of four main phases:

1. Set-up phase with the development of the participatory partnership, the conceptual framework, the study design and the recruitment and training of Community Researchers (CRs).

2. Fieldwork phase with sampling of sub-Saharan migrants in Morocco in the most relevant areas in Morocco where those migrants are residing through Respondent Driven Sampling and consequently the conduct of in-depth interviews on sexual health, sexual violence and perceived determinants with those migrants by the CRs.

3. Analysis phase in which the interviews were transcribed ad verbatim before being analysed with Nvivo 8 and interpreted, nuanced and validated at a public seminar at the University of Mohammed V in Rabat in presence of SAB, CRs and CAB. Policy, practice and research recommendations were also formulated at this point.

4. Dissemination of results and promotion of the policy, practice and research recommendations.

#### Set-up phase

The participatory partnership identified the cities of Oujda, Rabat, Casablanca and Tangiers as main locations where sub-Saharan migrants were residing. Subsequently, CRs needed to be recruited here as well as the in-depth interviews conducted. Inclusion criteria for eventual respondents and CRs were the same, namely being a female or male sub-Saharan migrant between 15–49 years old and living in irregular situation (refugee, asylum seeker or undocumented) in Morocco. Consequently, potential CRs were sought for through the networks of the CAB and were asked upon identification whether they could indicate other potential CRs. In spring 2008, 25 sub-Saharan migrants meeting the inclusion criteria were invited to an interview with SAB members and screened on necessary communication skills, potential research skills, empathic attitude, social engagement and leadership capacities. Eight women and four men descending from DRC, Cameroun, Angola, Central Africa, Nigeria and Ivory Coast were withheld. Four of them lived in Oujda, 3 in Rabat, 3 in Tangiers and 2 in Casablanca. They completed a 30 hours training to become CRs. This training addressed migration, human rights, sexual and reproductive health, several types of violence, gender, psychosocial education, intercultural communication, the study conceptual framework and epistemology and finally guidelines and exercises on conducting in-depth interviews in an empathic and ethically sound way. Different SAB and key CAB members gave these courses. All CRs received a certificate of attendance issued by the Belgian and Moroccan Universities and research associations involved. The preliminary interview guide, consisting of guidelines to address respondents, to obtain informed consent, to initiate the interview, to build the interview, to come to the key questions, to probe for more in-depth answers and to close the interview; was thoroughly discussed, exercised and adapted through consensus building with the CRs after which they were piloted before finalisation. We did this aiming to enhance the beneficial outcomes of the participatory research approach; secondly to maximise the match between ‘inner speech’ and the language used [48]; and, thirdly to optimise validity and reliability [49]. The interview guide was developed in French and English as these were the languages that most sub-Saharan migrants in Morocco master. We first questioned for respondents’ socio-demographic profile and their definition and perception of sexual health. Subsequently, we explored whether the respondents and/or close peers to them had experienced victimisation since having initiated their migration towards Europe and if they had, we probed for a detailed description of the victimisation acts, the context in which it happened and the consequences. Subsequently, we assessed their perception of risk and prevention factors and suggestions for action. This paper solely reflects on the latter two issues taking their socio-demographic profile into account. In parallel to this study, a Knowledge, Attitude and Practice survey on the role of the health care sector in prevention of sexual violence in sub-Saharan migrants was conducted among Moroccan health care workers, which is published elsewhere [50].

#### Fieldwork phase

It was set forward to conduct 50 interviews in both Oujda and Rabat and 30 in Casablanca and in Tangiers. Yet, when starting up the fieldwork phase in summer 2008, riots broke out in Oujda urging a lot of migrants to flee. As the security of our CRs could not be guaranteed and it appeared that most of the migrants had fled to Fes, it was decided to replace Oujda by Fes as research site. CRs were asked to conduct 12 to 15 interviews in their respective cities or surroundings with respondents meeting the above-mentioned inclusion criteria following the Respondent Driven Sampling rules. As Respondent Driven Sampling is specifically designed to research hidden networks of at risk populations in precarious situations [51-55] it served as a perfect sampling strategy for the aim of our research. How the waves would be constructed and what kind of primary and secondary incentives would be given to respondents and how the community researchers would be remunerated, was suggested by the participatory partnership and finally jointly decided upon with the CRs during the CR training. In a first wave, the twelve CRs served as primary seeds and searched for 5 peer migrants. Once identified, respondents were informed about the project objectives, the interview goals, the potential risks and measures taken to protect them from those risks. The respondents signed an informed consent before the interview and could withdraw at any point during the interview. The interviews were recorded while CRs simultaneously took notes on their interview guides. Upon completion of the interview, the respondents were given 100 MAD (about 9 €) as primary incentive for their participation as well as a coupon and a package with information on referral organisations providing different kinds of assistance to migrants, sexual and reproductive health issues and condoms. They were asked to identify a potential other respondent among their peers meeting the inclusion criteria and bring the CR into contact with this person. If they managed to recruit an additional respondent, the initial respondent could exchange his/her coupon for their secondary incentive of 25 MAD with the CR. These secondary wave respondents were then interviewed, received their primary remuneration and where then asked to do the same until every CR had conducted 12 to 15 in-depth interviews. Respondents could participate only once. In the four study sites 3 to 4 waves per CR were conducted. The CRs were coached by the Belgian and Moroccan coordinators who in turn passed along the four research sites and intensively supervised the CRs. In the first coaching session by the Belgian coordinator problems regarding sampling and mastering of the interview guide were discussed and addressed, and group debriefings as well as personal counselling was done addressing emotions arisen by the content of the interviews. In the second round the Moroccan coordinator addressed technical and administrative problems. In both rounds, the CRs handed over the interview guides they had completed together with the recordings and received their remuneration as agreed upon during the training, namely 200 MAD as remuneration for the interview and 50 MAD for phone and transport costs per conducted interview. Two Nigerian CRs dropped out during the last weeks of the interview period because of security threats they had received.

#### Analysis and dissemination phase

Eventually, 154 valid interviews were conducted in French or English. Interviews were considered valid when we had the completed interview guide, the notes taken by the CR, the signed informed consent and the recording of interview. They were checked for potential doubles as they were to be deleted but none occurred. All interviews were transcribed ad verbatim in the language they were conducted in both Morocco and Belgium after which they were entered into Nvivo 8. A first round of grounded coding was done by both the Belgian and Moroccan coordinator for consensus on main categories and procedures. Subsequently, the Belgian team continued the complete coding of all interviews and drafted a report with preliminary results that was presented at a public seminar at the University Mohamed V in Rabat in May 2009 in presence of the CRs, the SAB and enlarged CAB. The preliminary results were discussed, interpreted and validated through different thematic working groups in which policy, practice and research recommendations were formulated and dissemination and continuation strategies were decided upon. For a thorough description of this seminar and its outcome we like to refer to the report of the project [56] as well to our publication on the role of the Moroccan health care sector in prevention of sexual violence [50].

Further scientific analysis of the interviews and dissemination in peer-reviewed journals was assigned to the SAB. All quotes stem from the ad verbatim transcriptions of the in-depth interviews, yet the names are pseudonyms. Quotes that were originally in French were literally translated to English by the authors. The study protocol applied the WHO [57] & UNHCR [29] ethical and safety guidelines in researching violence. In line with the CBPR methodology, safety issues and project procedures where strongly debated and commonly decided upon by the CRs and the CAB, resulting in an ethical approval from the research community itself. Furthermore, ethical approval was granted by the Ethical Committee of the Ghent University Hospital. Finally, as is accustomed in Morocco, we informed the Ministry of Interior of our study protocol and of the approval of the partnership and subsequently negotiated ethical approval with each of the respondents through informed consent.

## Results

### Socio-demographic profile

In the summer of 2008, 154 valid interviews were conducted by community researchers in the cities of Rabat (46), Casablanca (30), Tangiers (31) and Fes (47). Table 1 sums up the most relevant socio-demographic characteristics of the respondents.

**Table 1:** Socio-demographic profile of respondents

**Total**	**154**	**100%**
**Gender**		
Female	60	38.96%
Male	94	61.04%
**Age (years)**		
< 18	11	7.14%
19 - 29	89	57.79%
> 30	53	34.42%
Unspecified	1	0.65%
**Country of origin**		
Democratic Rep. of Congo (DRC)	51	33.17%
Cameroun	25	16.23%
Congo Brazzaville	16	10.39%
Ivory Coast	15	9.74%
Mali	12	7.79%
Other sub-Saharan countries (14)	35	22.73%
**Residence status in Morocco**		
Asylum seeker	22	14.29%
UNHCR refugee	19	12.34%
Undocumented	107	69.48%
Other	6	3.90%
**Children in care**		
0	98	63.64%
1	26	16.88%
2	19	12.34%
3 to 5	10	6.49%
> 5	1	0.65%
**Religion**		
Christian	114	74.03%
Muslim	32	20.78%
Other	8	5.19%
**Attained education**		
Higher education	58	37.66%
Secondary education	67	43.51%
Primary school	20	12.99%
Did not attend school	5	3.25%
Other	4	2.60%
**Daily activities**		
*Country of origin*		
Paid employment	67	43.51%
Seeking employment	10	6.49%
Student	63	40.91%
No paid activity	14	9.09%
*Morocco*		
Paid employment	11	7.14%
Seeking employment	47	30.52%
Student	3	1.95%
No paid activity	92	59.74%
Unassigned	1	0.65%
**Arrival in Morocco**		
< 2 years	21	13.64%
2 - 5 years	102	66.23%
5 - 10 years	28	18.18%
> 10 years	3	1.95%

The majority were young, well-educated migrants who predominantly originated from the Democratic Republic of Congo, Cameroun, Congo Brazzaville, Ivory Coast and Mali. Most of them were living in Morocco for between two and ten years, but could not tap their skills and capacities due to their undocumented and unemployed status. A vast majority of respondents had completed secondary or higher education (81.16%). In addition, more than half of the respondents spoke at least one other language than their mother tongue (57.14%). Although few respondents were able to read and write Arabic, nearly half of them spoke some Moroccan Arabic (43.27%). Yet, more than 80% indicated to speak, read and write French, the second official language of Morocco. Furthermore, a large discrepancy is found between their occupational activities in their countries of origin and in Morocco. An overwhelming 90.25% declared having no paid activity or seeking employment. Also their housing conditions revealed to be poor: the majority (64.29%) lived in a single room they had to share with more than three other migrants. Half of the women lived with children, while men rather shared space with other adults. A third of the respondents had only access to toilets and could not take showers inside their accommodation.

### Experiencing violence

Among the 154 sub-Saharan migrants interviewed, 138 (89.61%) reported cases of sub-Saharan migrants being victimized by persons unknown to them either during their migration or in Morocco itself, while 16 did not report any violence experiences. Of those 138 respondents, 120 had been personally involved: 79 were physically and/or sexually victimized in person, while 41 were forced to witness how their partners, children, family members, friends or co-migrants were physically or sexually victimized in their presence. Eighteen other respondents only knew of sub-Saharan peers within their close relationship as relatives or friends who were victimized.

The 138 respondents described 230 independent cases of violence. The majority of those cases (132) took place in Morocco or at its borders. The most frequently mentioned cities were Oujda (74), Rabat (22) and Casablanca (12) while others indicated “in Morocco” (24). Outside Morocco, it were Maghnia or the Algerian-Moroccan border (24) - which refers to the route they take that necessitates a future passage through Oujda -, Algeria (17), Tamanrasset (8) and the desert between Mali and Algeria (8). In 26 other interviews, respondents described violence during their migration journey without indicating a specific place.

Analysing the 230 cases to the types of violence that occurred, we noted 548 single acts of violence which we classified in Table 2 according to types of sexual and gender-based violence as used in refugee and conflict settings [31]. This consisted of emotional (18%), physical (22%), sexual (45%) and socio-economic (14%) violence, indicating that most victims had to endure multiple forms of violence that were afflicted upon them in a combined way.

**Table 2:** Nature and scope of reported violence

Types of violence (*)	**Total acts (n = 548)**	**100%**
**Emotional/psychological violence**	**101**	**18.43%**
Confinement	36	6.57%
Threats	35	6.39%
*• of which with weapons*	*23*	*4.20*%
Verbal abuse	10	1.82%
Humiliation	2	0.36%
Combination	18	3.28%
**Physical violence**	**122**	**22.26%**
Singular non-life threatening	12	2.19%
Multiple non-life threatening	89	16.24%
Singular life threatening	1	0.18%
Multiple life threatening	6	1.09%
Killing	2	0.36%
Combination	12	2.19%
**Sexual violence**	**246**	**44.89%**
Rape	141	25.73%
*• of which gang and/or multiple rape*	*111*	*20.26*%
Sexual abuse	46	8.39%
Sexual harassment	33	6.02%
Sexual exploitation	24	4.38%
Sexual torture	2	0.36%
**Socio-economic violence**	**79**	**14.42%**
Stealing	73	13.32%
Refusal of first aid services	2	0.36%
Combination	4	0.73%
**TOTAL**	**548**	**100.00%**

(*)Classified according to the main types of sexual and gender-based violence as defined by UNHCR (2003).

#### Physical, emotional and socio-economic violence

The reported emotional violence regarded primarily confinement, threats – of which 23 with weapons - and racist verbal abuse.

“It happens sometimes when I go to the grocery store, there are Moroccans who insult you, call you ‘filthy nigger’, ‘slave’, they throw stones at you or if you are unlucky, they spit on you. And this everyday. You need a strong heart to walk the streets here in Morocco” Maryam, 25, female from Mali.

While socio-economic violence consisted in nearly all reported cases of stealing of money, resources and mobile phones and to a lesser extent of being denied access to basic services such as food or healthcare.

“Money, clothes, jewels, everything you have: they take it all and then they throw you towards the desert over there.” Vénédict, 30, male from Cameroun

As for physical violence, respondents reported primarily non-life threatening episodes of multiple physical violence, consisting mostly of severe beating and slapping.

“It happened here in Morocco during the refoulement, a young girl and a young boy got their legs broken by the Moroccan military. They refused to enter the truck used for the refoulement, and the soldiers hit them.” Célestine, 38, female from Rwanda

Yet, in a few cases the physical violence was life-threatening engendering a fatal outcome.

“He arrived at the Medina [in Casablanca] and a group of Moroccans came out of the Medina to attack my brother in law. They attacked him and hit him until he was half dead. Eventually, he was taken to the hospital where they tried to reanimate him to make him regain his health, but it was too late, he already died.” Elisa, 29, female from DRC

#### Sexual violence

Sexual violence was the most common form of violence with 246 sexual violence acts or 45% of all reported violence occurring in 184 of the 230 cases. The types range from sexual harassment (no physical contact) over sexual abuse (physical contact but no penetration) to rape and sexual torture (with penetration).

Sexual harassment episodes consisted mostly of cases in which the victims were publicly forced to undress or threatened with rape.

“They asked the women to undress in front of the men, just for their pleasure, and to dance. They were completely naked, in front of everybody. Without shame, just like that. It was horrible” Fabrice, 30, male, Ivory Coast

Sexual abuse consisted primarily of unwanted sexual touching and clothes being torn to reveal body parts, again mostly in group. Searches were sometimes used as pretexts for unwanted touching or penetration of private parts.

“There were cases of touching when we had controls and they were searching for money. They were putting, circulating their hands everywhere randomly.” Bene, 29, male from DRC

“There was already one touching my breasts. They said if my husband would not give them money, they would do whatever they liked with me” Vanessa, 21, female from Central Africa

“(…) they [a Nigerian gang at Oujda] beat us up and they were body-searching the girls, taking their clothes off and inserted fingers in their vaginas. The men who refused anal search, they were beaten to death.” Sylvestre, 28, male from Chad

Rape was the most common form of sexual violence (142/248). It predominantly consisted of gang rape (81/142), with at least two to more than ten perpetrators raping at least one victim at the same time; or of multiple rapes (30/142), where one or more perpetrators raped the victim(s) successively for a longer period of time. In 77 rape cases there was one single victim while in 65 other cases the respondents emphasized that victims were raped in group as well. The impossibility to resist or escape was stressed by both the victim(s) and co-migrants who were forced to watch.

“They brought us to an olive tree field, so they could do whatever they wanted with us. They raped us. They were seven. We were six girls, so you can imagine, it was not easy, and it’s life.” Beyoncé, 23, female from Cameroun

“He called three men who grabbed my head, they were holding me by the hair and they had sex everywhere, vagina, anus, breasts, and they put sperm all over me.” Agnès, 28, female from Rwanda

“It was the two girls that they took, and all six persons raped them, the two girls. (…) They put us on our knees with our hands on our head, but the mother of the two girls was put on her knees and with her forehead to the ground. (…) They hit the mother because she was crying for her children when she heard her children, she had, always, always, always, always you will shout, you will shout, always. When you hear how your child is weeping, you always, always, always shout. And that hurts, hurts badly.” Diane, 42, female from Burkina Faso

Sexual exploitation included forced prostitution and forced transactional sex in return for promised food, shelter, security or pass-through.

“He brought me to this man who gave me water, food, and a shower. Then he told me that this man helped many migrants, but that I would need to work like the others. I asked what I would do: he told me to be a prostitute and he would take the money.” Agnès, 28, female from Rwanda

“The two men took the youngster to their ‘tranquilo’ promising that they would help him find back the members of his community, help him to get to Rabat (…) The second night they tied him up and started to beat him everywhere until one Nigerian made him understand that as he did not have money he had to satisfy their sexual needs. The young guy refused, but with torture, he could not do anything as he was tied up.” Denzel, 19, male from Cameroun

### Identity of the victims and the perpetrators

Forty-five respondents (29.22%) reported to be sexually victimized themselves, 54 (35.06%) were forced to watch while their relatives, friends or co-migrants were sexually victimized in their presence and in 85 other sexual violence cases it were their peers, such as family, friends or acquaintances; who had been victimized independently of the respondent’s presence.

Given that so many sexual victimizations occurred in group, it is impossible to give exact numbers of gender proportion. However, the respondents clearly indicated in 202/248 of the sexual violence incidents (81.45%) that at least one girl/woman was victimized and in 93 incidents (37.50%) that at least one boy/man was victimized. For rape specifically, it regards female victimization in 121/142 rape incidents (85.21%) and male victimization in a 53 of the 142 reported rape incidents (37.21%).

“It’s like that they forced him to suck their penis in turn. While he was sucking, the other one penetrated him anally. Despite the shouting of the young guy, the men would not listen nor come to reason. One of the Nigerians used Vaseline and could rape the young guy penetrating him anally.” Denzel, 19, male from Cameroun

In a majority of cases, the victim’s age were not specified, although the respondents used “young”, “boy”, “girl” or “child” to describe non-adults in a third of the cases.

“They were six, they say black Moroccans, I cannot tell. One took out his sex, and he put the child in front here and them at the back [In front and at the back?] Yes, in the mouth. And when you see his big sex there it’s like the bottoms of a baby. The blood pours down the child and they don’t even have pity for that blood there, the other comes, and does it without a condom” Diane, 42, female from Burkina Faso

The origin of the perpetrators was mainly described as Moroccan (31.30%), Nigerian (26.09%), other sub-Saharan nationalities (14.78%), Algerian (10.46%) and “Arab” (5.21%). It is to be noted that when more description was given of the perpetrators, they can be identified as persons in authority the migrants were confronted with during their journey, as for example: soldiers (10.44%), police men (9.13%), guides (7.39%) and chairmen (4.4%). The military and police perpetrators were Moroccan or, when occurring at the border region, also Algerian.

“Because she had gone to enter Ceuta, and when they body-searched her, they raped her, it were 5 policemen, 5 Moroccan policemen.” Christian, 37, male from DRC

“Yes I remember the day that the Moroccan soldiers raped, we were victims of rape.” Sandrine, 22, female from Ivory Coast

“And then when we entered Tamanrasset the Algerian soldiers forced the mother to give her 2 girls (…) they raped them, and when they were ready they raped her son too, at his back” Fatoumata, 27, female from Mali

The chairmen/leaders and guides were sub-Saharan, predominantly Nigerian.

“It was at the time of the refoulement to Oujda, when they [Moroccan soldiers] set us free, we were looking for a way to come back to the city. On the way back we met a Nigerian chairman called Al Pacino and his gang. They stole from us again and they threatened to kill us, they beat us up and they were searching the girls, taking their clothes off (…)” Sylvestre, 28, male from Chad

“When we met the Nigerians in Oujda, they aggressed us. Just like that, for their pleasure” Fabrice, 30, male from Ivory Coast.

When it regarded chairmen/leaders, they frequently acted together with other disciples of his group having some authority, resulting invariably in gang rape. The perpetrators were then described as “gangs and their leader/chairman” (23.04%).

“Once we were resting at night, an armed group took us by surprise and they chose two women; their leader decided that only those two would be taken and then all the men - more than ten! - raped them” Elikia, 44, female from Congo Brazzaville

“At the border of Algeria with Morocco we saw Nigerians who were coming up to us saying we had to give them money. As we did not have any money the Moroccans said we will separate the girls. (…) What I have lived through with the girls who were with me, it is not possible. They abused us, thus they maltreated us, thus I don’t know how I could say it (…) and thus they really hurt us. They slapped us. And raped. (…) They were many, but I found myself with four on top of me.” Sarah, 24, female from DRC

Yet, also Moroccan citizens are identified as perpetrators of sexual violence.

“When we arrived in Fes, there were 2 Moroccans who passed by. We were 2 girls and 1 boy, they chased us. They just took the boy, and, thus the boy was raped in front of our eyes. They let us go after that saying that we were not allowed to tell otherwise they would kill us, they had knives.” Deiondre, 23, Female from Cameroun.

“It was in the Takadoum neighbourhood of Rabat (…) and when he was profoundly asleep of the drugs his roommate had put in his food, his roommate went to the Moroccans to say: ok, mission accomplished. The Moroccans lived in the same building and (…) they physically and sexually abused him (…) every time he woke up lying naked on his bed and he felt a liquid coming out of his anus” Deshawn, 31, male from Cameroun

“She took a taxi at Rabat Agdal station, (…) the [Moroccan] driver and his friend took her to the beach and on the big stones they took her by her wrists and enforced them brutally on her, they raped her, (…) first the one, then his friend took also advantage, he did the same” Malika, 25, female from Niger

In some cases it also concerned other irregular migrants in distress, and once again the Nigerian origin was frequently stressed.

“The young guy was deported back to the border by the police. He went to Oujda and found himself there, alone without money (…) two Nigerians in need made a sexual object of this young guy” Kwambe, 28, male from Cameroun

### Consequences of sexual violence

In 124 interviews, respondents reported a wide range of consequences following sexual violence episodes. They most often comprise of a combination of emotional and physical consequences, which are in some cases accompanied by sexual, reproductive and/or socio-economic effects. Some of them stem directly from the sexual victimization while others are induced by those direct results and emerge later on. Again, the powerlessness of both the victim(s) and co-migrants who were forced to watch was emphasized as remaining very disturbing and hard to cope with at the long run.

#### Emotional/psychological consequences

The sexual victimization triggered a wide range of affective consequences most commonly described as “shame”, “restlessness”, “not able to speak”, “fear” and “emotional breakdown” which regularly resulted in further impairment of their relationships with their partners, families and communities.

“she was so ashamed that everyone knew she had been raped, that she locked herself up in her room all day long.” Binéka, 22, female from Congo Brazzaville

“*You can see she is not at peace, it looks like she hurts, and it’s hard for her to talk about it and when she does she always cries. What she regrets is that they were many but she does not remember how many. She says that she could feel nothing but pain for one month, and that it’s still in her mind*.” *Amandine, 17, female from DRC*

#### Physical consequences

Reported physical consequences of violence were predominantly temporary. Those included wounds, loss of consciousness or blood, belly pains, difficulty to walk or sicknesses. However, in 10 cases the consequences were permanent, as on-going pain and injuries such as vaginal and anal tearing, physical impairment or even fatal consequences in 6 cases.

“The day after [the 2 women were raped], our men transported them but they could not talk, then we had to leave them behind because the men could not carry them anymore. Given where they were, they certainly died. It hurts, because we walked together.” Elikia, 44, female from Congo Brazzaville

“See my hand? I miss two fingers.” Wamba, 34, male from DRC

#### Sexual and reproductive consequences

In 51 cases, precisions were brought as to sexual and reproductive consequences of violence. Reproductive consequences included unwanted pregnancies following rapes. Abdominal pains, STIs and HIV/AIDS infections were mentioned as sexual consequences of the victimization.

“Some of them became pregnant, others had STIs, we all know these cases” Adjoussou, 40, male from Ivory Coast

#### Socio-economic consequences

Socio-economic consequences were cited in 22 cases. They covered mainly testimonies of social exclusion and a lack of support structures for victims in Morocco. Some victims decided to go back to their country of origin if they could or simply wished to leave Morocco because of their experiences during migration.

“My child is one year and four months now, I became a mother too young. My parents do not trust me anymore, and the Congolese community thinks I’m a whore, an easy girl. I would like to leave Morocco to forget everything that happened here.” Grace, 17, female from DRC

### Perceived risk and prevention factors

#### Risk factors

Respondents were subsequently asked which factors they perceived as potentially putting a person at risk for sexual violence, and to determine which ones they would identify as the three most important ones. All but two respondents answered those questions.

Respondents cited risk factors primarily at the societal and public policy level, stating that these affect all other socio-ecological levels. The legal status of migrants was considered a major risk factor and it is the preferred answer for both women and men and the predominant answer for young people aged fewer than 30. Answers given in that category particularly pointed at the situation of undocumented migrants.

“And when they arrest you, they will start to, euh, maltreat you, shout at you, do whatever they want with you, just because you don’t have papers. (…) We don’t have any papers. The papers that UNHCR gave us maybe? The authorities here don’t value those UNHCR papers, they don’t consider them.” Vénédict, 30, male from Cameroun

Furthermore, they indicated the dangerousness of the physical environment in which migrants live, highlighting the absence of laws and protection in specific places and notably around borders. The refoulement -the fact of being arrested and deported to Oujda or across the border- was cited by many as increasing risk of victimization.

“It’s because of the surroundings. The fact that we came, the route we took, it’s the surroundings, the place, the place where there are gangs, so in these places, you cannot flee. You can’t flee from there.” Fabrice, 30, male from Ivory Coast

A low socio-economic position was also identified as risk factor. They defined this as financial hardship (113 occurrences), the lack of employment, bad accommodation and poor opportunities within the Moroccan society. In addition, poverty in both the country of origin as well as in Morocco was stressed as risk factor; the former for forcing people to migrate in dangerous conditions and the latter to maintain them in a vulnerable state.

Respondents also identified risk factors at an individual level on both the victims’ and the perpetrators’ sides, yet to a much lesser extent. A number of respondents stated that the behaviour of some migrants could put them at risk for sexual violence. Answers given in this direction particularly targeted clothing, looks and attitudes of victims. Risk factors for committing sexual violence included considerations on the lack of sexual activity and the will to hurt others.

#### Prevention factors

All but eight respondents (4 M and 4 F) indicated means by which they think sexual violence against sub-Saharan migrants in Morocco and its borders could be prevented. Questions in this heading asked them to give their views on prevention factors both within sub-Saharan communities and the Moroccan society.

A fourth of the identified prevention factors addressed the individual and interpersonal level. Respondents insisted on the need to avoid a number of situations considered as risky, such as engaging in contacts with strangers or going through dangerous places. Being documented and employed was also perceived as preventive, as was the presence of a strong social network. Finally, answers indicated that awareness and knowledge on risk and prevention could prevent further sexual violence to happen.

Most respondents identified potential prevention actions at the organizational and community level (551/1047 suggestions). Again here, they mainly focused on knowledge transfer and suggested a wide range of channels as schools, associations, religious structures, health services and media for spreading sensitization campaigns on sexual violence. Raising awareness was also perceived as creating a sort of safety net protecting migrants from (re)victimisation. Finally, they believe the migrant communities should be empowered and organised on the matter which could enable them to subsequently lobby Moroccan authorities.

“If each of our communities could get organized and have a leader; those leaders could then lobby the Moroccan authorities to put an end to the assaults and rapes, even if the Moroccan authorities did not ask for us to come here. The communities could create networks to sensitize people in different neighbourhoods.” Koffi, 35, male from Congo Brazzaville

Prevention factors at macro level (246/1047 suggestions) targeted a wide range of policy changes. They firstly suggested that the Moroccan borders should be better secured and facilitate migration. Secondly, respondents emphasized that the Moroccan government should consider granting documents to more migrants in order to stabilize their status within the country and to avoid situations of refoulement. Thirdly, they requested to apply laws that punish perpetrators of sexual violence as they believed this could refrain potential perpetrators from acting out. Making use of human rights to advocate for such policies was suggested by some.

“[Perpetrators] must be condemned and very severely. Today there is impunity” Mamadou, 26, male from Ivory Coast

“What could contribute most [to prevention] is public action” Miezi, Female, 24, Congo Brazzaville

Although the respondents suggested so many actions to take, 16% of them stated that preventing sexual violence would encounter significant obstacles. They highlighted the difficulty for migrants to identify moments at which they could be at risk for sexual violence and insisted that information presents the only realistic way of preventing sexual violence.

## Discussion

### Nature and scope of violence

Our respondents stressed that violence in general and sexual violence in particular has just become an unavoidable part of the journey. Our findings support this perception as they demonstrate that multiple physical, psychological, sexual and socio-economic victimizations of sub-Saharan migrants in Morocco and at its borders are a common phenomenon and that almost half of this victimization is of sexual nature. This confirms previous tentative findings regarding migrants’ sexual victimization in Morocco [1,3,41] and might be reinforced by the fact that many migrants had already been sexually victimized in their countries of origin, and that sexual violence was for some of them part of their decision to migrate [3,15]. The omnipresence of sexual violence within sub-Saharan migration leads to a ‘normalization’ of sexual [15] and other types of violence [9], and could be linked to what in literature on sub-Saharan migration has been identified as ‘hardship as an initiation rite’ [9]. The fact of placing this in a context of cultural initiation rites, in which one endures a hardship during a certain period of time in order to get to a next phase of life with an enhanced state of being, could be interpreted as a coping strategy of the sub-Saharan communities. Trying to commonly interpret an adversity and subsequently adapt to it as a community, is as such a healthy reaction in community resilience [58]. Yet, the sexual violence reported here is of such invasive and destructive nature inducing severe long lasting ill-health consequences in a context where victims are not granted access to official health care but are dependent on rare NGO-medical and social support upon sexual victimization [50], that it can hardly build a person. On the contrary, the way the sexual victimization is performed bears many similarities with sexual violence as a weapon of war, which, by definition, has the purpose of destabilizing or extinguishing a group of people, because they belong to that group of people [29]. Even though it would take us too far to conclude that the purpose is to destabilize or extinguish sub-Saharan migrants, they are targeted here because they are undocumented sub-Saharan migrants without legal protection. Our results confirm that, given the lack of official authorities at many migration ‘hubs’ and given their undocumented status outside the ECOWAS region, the migrants are in a very vulnerable situation risking more victimization, exploitation or deportation when they would try to report or fight against it [9]. Furthermore, as in sexual violence as a weapon of war [59-61], gang rape is the most common form of reported sexual violence, and if migrants are not victimized personally, they are often forced to witness how their relatives or co-migrants are victimized in their presence, which is equally traumatizing. The respondents emphasized how heavy the burden is to live with the fact that they could not interfere or resist just because they were so many. Furthermore, the gang rapes are often perpetrated by either Moroccan and/or Algerian officials (soldiers, police) or gangs of Nigerian chairmen and other sub-Saharan migrant leaders who are self-identified migration professionals who installed them there where official authorities are lacking or refrain from interfering, and where migrants do have to pass on their migration route to and through Morocco. This is especially the case in the border region of Algeria (Maghnia) and Morocco (Oujda) where most of the reported sexual victimization took place. Where money and other material belongings cannot be confiscated anymore as bale for passage, migrants have to pay with their bodies. It is often the young women and men of the group and sometimes even the children who are picked by the perpetrators to be sexually victimized in return for the group’s passage. The young and child migrants symbolize the hope for a better future, a purpose of migration. Yet, these bodies which bear the reason to live for the migrants, are what Agamben G. (1998) called “stripped to the bare life” at different levels. First of all literally by the perpetrators, yet also figuratively by all authorities directly and indirectly involved, who treat them as if their lives are devoid of value, “unworthy of being lived” [62]. As the perpetrators bear the local authority and sovereignty, and the victims are undocumented or have UNHCR papers which still need official recognition and subsequent protection by the Moroccan state until today, and as neither the Moroccan, Algerian nor European governments interfere; it seems that the perpetrators can proceed in mere impunity.

### Treatment, prevention and response

The consequences of sexual violence listed by respondents were numerous and often combined long lasting sexual, physical, mental and socio-economic consequences which are again in line with what is found in victims of sexual violence as a weapon of war [60,63-65]. This confirms the need for desirable [47], holistic and multilevel prevention [29,66] and response actions to sexual violence targeting sub-Saharan migrants in and around Morocco. Our respondents told that they relied on NGOs to seek assistance, identifying organizations such as the Red Cross or Doctors without Borders (MSF) as sole care providers for treatment of sexual violence victims. This is an unsustainable situation and ignores the migrants’ right to health care. The lack of possibilities for these populations to find help within public health services is confirmed by the simultaneously conducted study on the views of healthcare practitioners on sexual violence against Sub-Saharan migrants in Morocco [50]. The interviewed health care workers highlighted the general weaknesses of the Moroccan public health system in terms of response to sexual violence, and additional difficulties met by migrants at the entry of health services. It is therefore not surprising that migrants also cite NGOs and associative networks as a prime medium for prevention of sexual violence. Although many pointed at the role Moroccan authorities should have in preventing and responding to sexual violence, they stated that this role was not carried on yet. Even when Moroccan security or police forces do not perpetrate violence, they might discourage migrants from seeking help. Among our respondents, none indicated that the victims reported the abuse to the authorities; on the contrary, many migrants stressed that their status prevented them from disclosing sexual violence cases, as this could lead to deportation. Additionally, their undocumented status in Morocco further hampers the victims in finding employment, with puts them subsequently at risk of more victimization, increasing their vulnerabilities [15]. Thus, a “life first” approach [67] in which needs are translated into rights demanding not only survival but also well-being and flourishing is urgently to be negotiated.

### The role of legal and policy frameworks

Our results confirm earlier findings [13,14] that the legal and policy framework on migration in Morocco do not protect undocumented migrants from being sexually victimised and even fuel uncertainties and violations of rights in the field [1]. Moreover, we argue that the externalisation of EU migration policies [13,24] contributes to the vulnerability of these migrants. The perpetuate use of the ‘transit migration’ concept participates in the invisibility of violence committed against undocumented migrants in border areas (Algeria-Morocco, Morocco-EU) by allowing all those countries involved as well as the EU as such to refuse accountability for these acts and putting the responsibility to a country across another border who can do the same up till the root countries the “migrants” originate from. Consequently, the so-called ‘transit countries’ have very little incentive to develop a rights-based approach towards migrants and to implement dedicated structures and services. In this context, their victimisation nearly seems “collateral damage” of the ENP: protection nor response to these victimisations are taken into consideration and migrants’ health is not considered a human right, let alone a public health issue. Moreover, Doctors without Borders recently reported that the NGO received 697 victims of sexual violence between 2010 and 2012, most of them reporting both multiple and gang assaults [68]. The sexual victimization thus continues in impunity, confirming that the lives of these migrants are politically not cared for, not by self-identified local authorities, not by Morocco, not by the EU and not at global level. Yet, in September 2013, the Moroccan government announced to create an asylum seeker status and judicial guarantees for the rights of the undocumented migrants entering into force by mid 2014 [69].

### Participatory approach

The application of the CBPR method and its principles in all phases of the project is described in detail in the methods section of this paper. An overall evaluation of what the project had brought about in the research community was done at the participatory end seminar. For a thorough description of this seminar and its outcome we like to refer to the report of the project [56] and for all policy, practice and research recommendations made, we refer to our publication on the role of the Moroccan health care sector [50]. Yet, we like to emphasize here that the project has yielded overall positive effects and outcomes to all parties involved. First, the respondents stressed that they welcomed the topic of the research and thanked the team for being genuinely interested in their lives, which confirms earlier findings on sexual violence research [70]. Second, the CRs felt their knowledge on the topics at hand as well as their communication skills were strongly improved as a result of being a CR. It is however noteworthy that although all CRs participated in the same training, and interview guides were commonly developed and agreed upon in both French and English, we cannot guarantee that their epistemological perspective while conducting the in-depth interviews might have differed slightly from the ones of the scientific advisory board. These elements might induce some biases in the data and thus a limitation to our study. Furthermore, although at first, several were worried to become key persons in the migrant community on issues of violence and sexual health, at the end all -but the two Nigerian CRs who quit the project in the last weeks of fieldwork due to security threats they had received- felt strengthened as a person, had a better self-esteem, and for some of them this even resulted in job offers majorly affecting their legal status and socio-economic position. For others, their legal status did not change, which urged them to continue their irregular migration to Europe. Also at the side of the organisations, institutions and policy makers which were invited to the CAB, an initial reluctance linked to security reasons of working with irregular migrants, and even scepticism on feasibility; changed into a perseverant engagement of a growing group of organisations, institutions and policy makers who still try to take the issue further in Morocco and in Europe today. This already resulted in the implementation of several of the project’s recommendations in changing formations, while others are still being lobbied for at national and international political instances or are submitted for project funding. For the principle researchers, it has proven that CBPR is scientifically a valuable but very time-consuming method which might challenge the established ways of conducting research yet resulting in a much more comprehensive understanding of the topic of research, which is in line with CBPR literature [44].

## Conclusion

Sub-Saharan migrants are at high risk of multiple sexual victimizations in and around Morocco. If not being personally victimized, many are forced to witness how their relatives or co-migrants are victimized by Moroccan or Algerian officials and/or by gangs of sub-Saharan chairmen who function as unofficial yet rigorous migration professionals. The ways in which this sexual violence is performed bears many similarities with sexual violence as a weapon of war. While many sub-Saharan young and child migrants pay for the passage of a group with their bodies, destroying their personal health and destabilizing the ones of those migrants who have to witness the victimization, it does not seem that their lives are politically cared for by any official authority. Comprehensive cross-border and multi-level prevention actions are thus urgently called for. Respondents link risk factors mainly to their undocumented and unprotected status and suggest that migrant communities set-up awareness raising campaigns while legal and policy changes enforcing human rights, legal protection and human treatment are lobbied for at different levels. Given the European Neighbourhood Policy, we deem it paramount that the European Union takes up its responsibility, drastically changes migration regulation into one that upholds human rights beyond the level of survival and enforces the Moroccan and all other authorities involved to restore migrants’ lives worthy to be lived again.

## Abbreviations

AIDS: Acquired Immunodeficiency Syndrome; CAB: Community Advisory Board; CBPR: Community Based Participatory Research; CRs: Community Researchers; EC: European Commission; ECOWAS: Economic Community of West African States; ENP: European Neighbourhood Policy; EU: European Union; HIV: Human Immunodeficiency Virus; RDS: Respondent Driven Sampling; SGBV: Sexual and Gender-Based Violence; STI: Sexually Transmitted Infections; SV: Sexual Violence; UNHCR: United Nations High Commissioner for Refugees.

## Competing interests

The authors declare that they have no competing interests.

## Authors’ contributions

As coordinator of the project, IK was responsible for project management, research conception, design, acquisition of data, analysis and interpretation of data as well as for the manuscript from draft to publication. AD as local field coordinator & AM as expert/project partner gave substantial intellectual contribution to research conception, design, acquisition and interpretation of data while JK conducted in close collaboration with IK the complete analysis and interpretation of data from input to drafting the preliminary research reports. NV, KR and MT participated in the scientific advisory board and thus had decisive input in every phase of the research project. They all revised the draft manuscript critically and approved the final version.
